# GalaxyRefine: protein structure refinement driven by side-chain repacking

**DOI:** 10.1093/nar/gkt458

**Published:** 2013-05-21

**Authors:** Lim Heo, Hahnbeom Park, Chaok Seok

**Affiliations:** Department of Chemistry, Seoul National University, Seoul 151-747, Korea

## Abstract

The quality of model structures generated by contemporary protein structure prediction methods strongly depends on the degree of similarity between the target and available template structures. Therefore, the importance of improving template-based model structures beyond the accuracy available from template information has been emphasized in the structure prediction community. The GalaxyRefine web server, freely available at http://galaxy.seoklab.org/refine, is based on a refinement method that has been successfully tested in CASP10. The method first rebuilds side chains and performs side-chain repacking and subsequent overall structure relaxation by molecular dynamics simulation. According to the CASP10 assessment, this method showed the best performance in improving the local structure quality. The method can improve both global and local structure quality on average, when used for refining the models generated by state-of-the-art protein structure prediction servers.

## INTRODUCTION

The structure of a protein can be predicted accurately from its sequence by template-based modeling when the sequence identity is sufficiently high (e.g >30%) (1,2). However, even at a high sequence identity, side-chain structure may be less accurate than the backbone structure, whereas at a lower sequence identity, predicted structures may have significant errors in both side-chain and backbone structures. Although *ab initio* protein structure predictions from sequences are notoriously difficult (3,4), *ab initio* refinement starting from a reasonable initial model structure is expected to be less difficult. Successful refinement can increase the applicability range of template-based models by providing more precise structures for functional study, molecular design or experimental structure determination (5,6).

Since 2008, various refinement methods have been tested in the refinement category of the community-wide protein structure prediction experiment Critical Assessment of techniques for protein Structure Prediction (CASP) (5,6). Several methods were shown to improve the initial model structures (7–12). Consistent improvements in such refinement experiments is more difficult than the typical refinement tests performed on lower quality initial structures, as the initial structures are selected from the best models submitted by CASP predictors, which have been already refined by other prediction methods (6).

In this article, we present a new model structure refinement web server called GalaxyRefine that has shown consistent improvement in CASP10, the most recent CASP held in 2012. GalaxyRefine first rebuilds all side-chain conformations and repeatedly relaxes the structure by short molecular dynamics simulations after side-chain repacking perturbations. Interestingly, this method can improve global and local structure quality. The method can improve global and local structure accuracy as well as physical correctness in 59, 67 and 79% of the CASP10 refinement category targets when measured by GDT-HA (13), GDC-SC (14) and MolProbity score (15). This method has been assessed to be more successful in refining the local structure and side-chain quality than any other methods tested in CASP10. GalaxyRefine also provides four additional models generated by relaxation simulations after larger perturbations on secondary structure elements and loops, resulting in larger changes from the initial model structure. GalaxyRefine can improve the models generated by state-of-the-art structure prediction servers such as I-TASSER (16) and ROSETTA (17) when tested on the server models submitted in CASP10.

## THE GALAXYREFINE METHOD

GalaxyRefine first rebuilds all side-chains by placing the highest-probability rotamers (18), starting from the core and then extending to the surface layer by layer. On detecting steric clashes, rotamers of the next highest probabilities are attached. After attaching all side chains, the number of neighboring C_β_ atoms is counted around each side chain, and the initial side-chain conformation is recovered if the number deviates from the canonical distribution for the amino acid under the same degree of surface exposure.

The model with the rebuilt side chains is then refined by two relaxation methods, a mild relaxation and an aggressive one. The lowest energy model of 32 models generated by the mild relaxation is returned as model 1, and four additional models closest to the four largest clusters of 32 models generated by aggressive relaxation are returned as models 2–5. Both of the methods are based on repetitive relaxations (22 and 17 for mild and aggressive relaxations, respectively) by short molecular dynamics simulations (0.6 and 0.8 ps for mild and aggressive relaxations, respectively) with 4 fs time step after structure perturbations. Structure perturbations are applied only to clusters of side chains in the mild refinement, whereas more forceful perturbations to secondary structure elements and loops are applied in the aggressive refinement. The triaxial loop closure method (19–21) is used to avoid breaks in model structures caused by perturbations to internal torsion angles.

The energy functions used for the two relaxation methods are linear combinations of a physics-based energy function complemented by database-derived terms and a harmonic restraint energy derived from the given initial model structure. The relative weight of the restraint energy to the physics-based energy for the mild relaxation is five times larger than that for the aggressive relaxation. The physics-based energy function contains CHARMM22-based molecular-mechanics bonded energy terms (22), Lennard–Jones interaction energy, Coulomb potential energy, FACTS solvation free energy (23) and solvent accessible surface area energy, whereas the database-derived energy function contains hydrogen bond energy (24), dipolar-DFIRE potential energy (25) and side-chain and backbone torsion angle energy (26).

### Performance of the method

**Figure 1.**
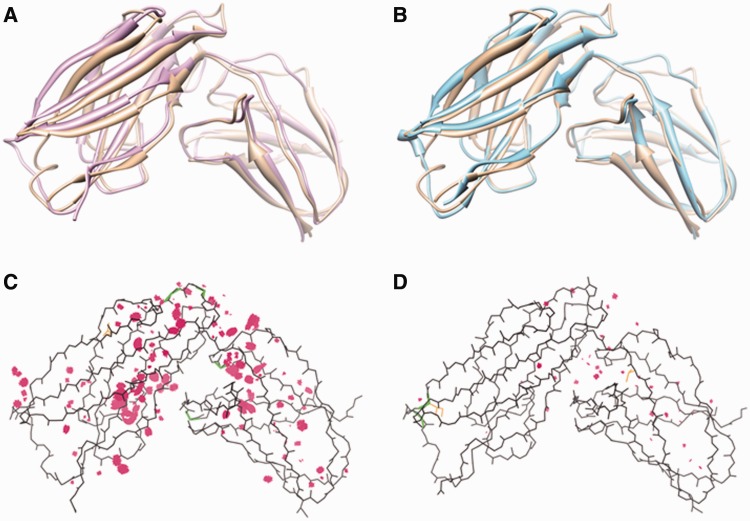
Refinement results for a CASP10 target TR681. (**A**) The initial structure (pink, GDT-HA = 57.6) and (**B**) the refined structure (cyan, GDT-HA = 64.1) is shown superimposed to the experimental structure (brown). Multi-criterion kinemage of (**C**) the initial structure (MolProbity score = 2.90) and (**D**) the refined structure (MolProbity score = 2.06). MolProbity highlights steric clashes as pink spikes, poor rotamers as gold side-chains and Ramachandran outliers as green lines.

The GalaxyRefine method has been extensively tested on (i) the refinement category targets of CASP8 (5), CASP9 (6) and CASP10 (53 proteins), (ii) Zhang-server (I-TASSER) models (84 proteins) (11) and (iii) ROSETTA server models (69 proteins) (17) for CASP10 template-based modeling targets and (iv) FG-MD benchmark set targets (147 proteins) (8). The test results in terms of improvement of model 1 (and the best refined model out of model 1–5) over initial input models for backbone structure accuracy measured by GDT-HA (13), side-chain structure accuracy measured by GDC-SC (14) and physical correctness measured by MolProbity score (15) are summarized in Table 1. The GalaxyRefine server shows average improvement in all test cases except for the MolProbity score of ROSETTA models, which have exceptionally good MolProbity scores. Although GalaxyRefine can improve GDT-HA and GDC-SC for all test sets, the average improvements are small (<1 and <3%, respectively), suggesting the necessity for further improvement in this field. Improvement in MolProbity score is relatively larger with an average improvement of 0.6 (from 2.58 to 1.96). Typical MolProbity scores for experimental structures are in the range of 1–2. A successful refinement example is illustrated in Figure 1.

**Table 1.** GalaxyRefine test results for model 1 (and the best model out of model 1–5 in parentheses)

Test set		Number of targets	Mean improvement/Median improvement/Percentage of improved targets		
			GDT-HA	GDC-SC	MolProbity score
CASP refinement category targets	CASP8	12	0.57/0.26/50 (1.45/0.63/67)	3.43/3.02/83 (4.07/3.07/83)	0.99/1.14/100^a^ (1.25/1.27/100^a^)
	CASP9	14	0.78/0.72/64 (2.19/1.22/93)	0.62/-0.05/43 (1.09/0.87/57)	0.62/0.44/71 (0.84/0.71/71)
	CASP10	27	0.08/0.63/59 (1.06/1.52/67)	1.10/1.36/67 (1.96/2.67/67)	0.70/0.80/79 (1.50/1.47/96)
	All	53	0.38/0.63/59 (1.45/1.19/74)	1.50/0.95/64 (2.21/2.36/68)	0.74/0.86/82 (1.26/1.37/90)
CASP10 server models	I-TASSER^b^	84^c^	0.41/0.44/66 (1.40/1.13/76)	2.52/2.22/87 (3.42/3.08/92)	0.69/0.73/98 (1.01/1.06/99)
	ROSETTA^d^	69^c^	0.45/0.49/64 (1.33/0.93/75)	0.67/0.59/64 (1.47/1.45/73)	−0.03/−0.14/26 (−0.01/−0.05/44)
FG-MD benchmark set		147^c^	0.61/0.81/65 (1.80/1.69/80)	1.74/1.24/75 (2.78/2.47/87)	0.89/ 0.92/100 (1.18/1.16/100)

^a^Initial structure for the target TR476 has no side-chain coordinates; therefore, it is excluded in the MolProbity analysis.

^b^Zhang-server models submitted for the CASP10 TS category targets,

^c^Non-oligomeric targets with TM-score (27) >0.5 and no severe crystallographic contacts.

^d^ROSETTA-BAKER server models submitted for the CASP10 TS category targets.

## THE GALAXYREFINE SERVER

### Hardware and software

The GalaxyRefine server runs on a cluster of 4 Linux servers of 2.33 GHz Intel Xeon 8-core processors. The web application uses Python and the MySQL database. The refinement method implemented in the GALAXY program package (28–31) is written in Fortran 90. The Java viewer JMol (http://www.jmol.org) is used for visualization of predicted structures.

### Input and output

**Figure 2.**
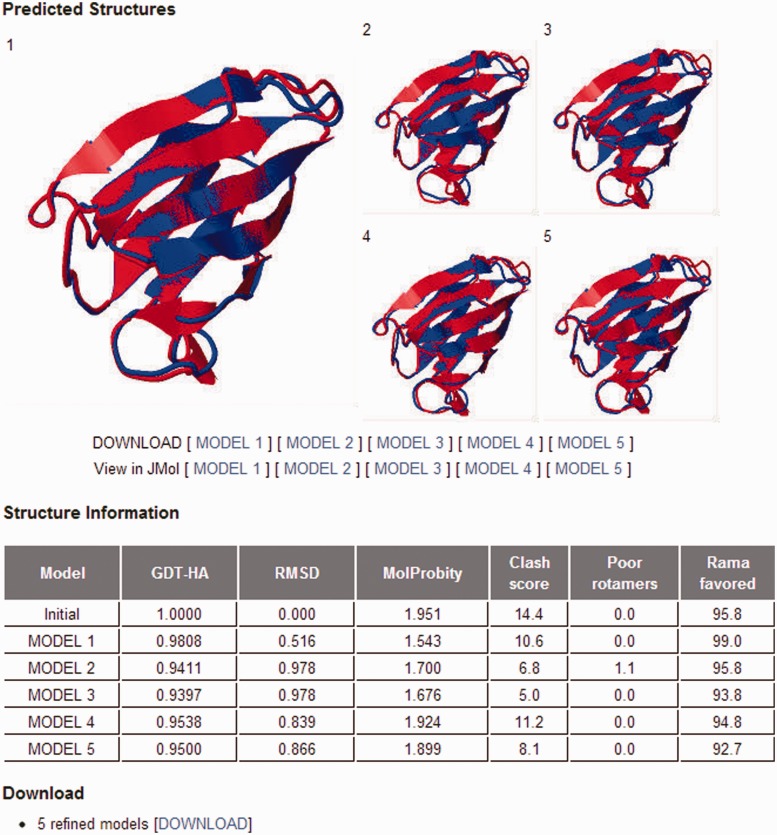
GalaxyRefine output page. The five top-ranking models are shown in static images, and they can also be viewed using the Jmol structure viewer. The structure changes relative to the initial model in terms of GDT-HA, RMSD and MolProbity score are presented in a separate table. Three components of the MolProbity score, namely, the number of atomic clashes per 1000 atoms, the percentages of rotamer outliers and Ramachandran favored backbone torsion angles, are also reported in the table.

The only required input is a single-chain protein structure without internal gap in the PDB format. The expected run time is generally 1–2 h. Five refined models can be viewed and downloaded from the website (Figure 2). Information on structural changes obtained by the refinement of the input structure is provided in terms of GDT-HA, RMSD and MolProbity score in a separate table.

## CONCLUSIONS

GalaxyRefine is a web server for protein model structure refinement that is particularly successful in improving local structure quality as demonstrated by the tests on CASP refinement category targets and CASP10 server models. On average, it shows moderate improvement in backbone structure quality. The server may be used to refine model structures obtained from available structure prediction methods, including the current best template-based modeling servers.

## FUNDING

National Research Foundation of Korea funded by the Ministry of Education, Science and Technology [2012-0001641, 2011-0012456 and 2012M3C1A6035362]. Funding for open access charge: Seoul National University.

*Conflict of interest statement.* None declared.
